# Supplementary material for: The interactome of intact mitochondria by cross-linking mass spectrometry provides evidence for coexisting respiratory supercomplexes
Source: Mol Cell Proteomics. 2017 Dec 8;17(2):216–32. doi: 10.1074/mcp.RA117.000470 (PMC5795388; doi:10.1074/mcp.RA117.000470)
Supplement: Supplemental Data [file supp_RA117.000470_133922_0_supp_23978_fzffwf.zip › spectra_annotation/mito_spectra_annotation/87-1-9-1-3-1.pdf]

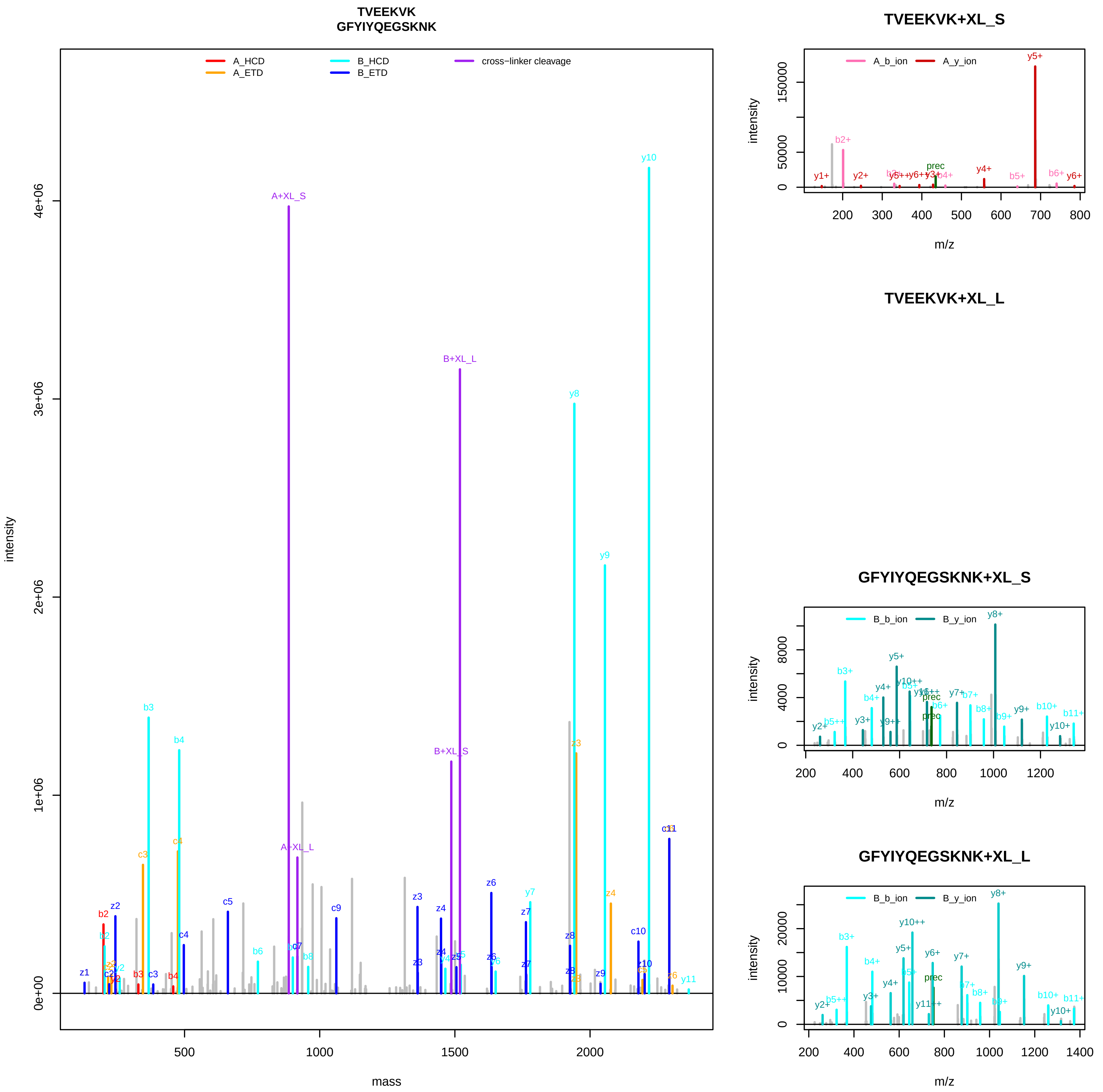

TVEEKVK
GFYIYQEGSKNK
A_HCD
A_ETD
B_HCD
B_ETD
cross−linker cleavage
intensity
mass
0e+00
1e+06
2e+06
3e+06
4e+06
500
1000
1500
2000
A+XL_S
B+XL_L
B+XL_S
A+XL_L
y10
y8
y9
b3
b4
c3
c4
z3
z4
c11
c10
z6
y7
z7
z8
z9
c5
c9
z2
b2
c2
z1
b6
y11
TVEEKVK+XL_S
A_b_ion
A_y_ion
intensity
m/z
0
50000
150000
200
300
400
500
600
700
800
y5+
b2+
prec
y4+
y1+
y2+
b5+
b6+
y6+
TVEEKVK+XL_L
GFYIYQEGSKNK+XL_S
B_b_ion
B_y_ion
intensity
m/z
0
4000
8000
200
400
600
800
1000
1200
y8+
y5+
b3+
y10++
y4+
b4+
y7+
b7+
b8+
y9+
b10+
b11+
y10+
y2+
y3+
prec
GFYIYQEGSKNK+XL_L
B_b_ion
B_y_ion
intensity
m/z
0
10000
20000
200
400
600
800
1000
1200
1400
y8+
y10++
b3+
y5+
y6+
y7+
b4+
y4+
y3+
y2+
y9+
b10+
b11+
y10+
b8+
prec
